# Values-Based Practice: A New Partner to Evidence-Based Practice and A First for Psychiatry?

**DOI:** 10.4103/0973-1229.40565

**Published:** 2008

**Authors:** K. W. M. Fulford

**Affiliations:** * *Professor of Philosophy and Mental Health, University of Warwick and Member of the Philosophy Faculty, Honorary Consultant Psychiatrist, and Fellow of St Cross College, University of Oxford, United Kingdom*

## Introduction

Recent years have seen the emergence of values-based practice as the “philosophy into practice” cutting edge of the new interdisciplinary field of philosophy of psychiatry.

In this editorial I start with a brief outline of the relationship between values, values-based medicine, and values-based practice in clinical decision-making. I then describe a number of key developments in policy, training, and research in values-based practice in the UK and internationally. Finally, in a brief concluding section, I indicate the way in which values-based practice, along with related developments in the wider field of philosophy of psychiatry, is showing the way forward for a closer and more effective partnership between values-based and evidence-based approaches, not just in psychiatry but in medicine as a whole.

## What Are Values? And What Is Their Relevance To Medical Decision-Making?

Perhaps the most familiar values in medicine today are ethical values. Certainly, few would doubt that ethical problems are widespread in medicine. However, values encompass much more than ethics. They include ethical values, but also values of many other kinds—preferences, needs, hopes, expectations, and so forth—and all these are important areas of values in medicine.

A helpful definition of values is provided by Sackett *et al*., in their training manual on evidence-based medicine: “By *patient values* we mean the unique preferences, concerns and expectations each patient brings to a clinical encounter and which must be integrated into clinical decisions if they are to serve the patient” (Sackett *et al*., 2000, page 1; emphasis in the original).

So, what is the common feature of values? What is it that makes all these very different kinds of values relevant to medical decision-making? The nature of values is one of those topics that has been widely debated by philosophers for over 2,000 years! But an important feature of values, and one by which they are at least partly defined, is that, as a former White's Professor of Moral Philosophy in Oxford, R. M. Hare, put it, they are “prescriptive” or “action guiding” (Hare, 1952; 1963).

It is this action-guiding feature of values that makes them not only relevant to, but also inseparable from, clinical decision-making. Values are of course explicitly present in some areas of medical decision-making, for example in cost-benefit analyses as the basis of the development of clinical guidelines (Brown *et al*., 2005). But Hare's point is that, whether explicit or not, values as well as evidence underpin *all* decisions, including, as those working on decision theory in medicine have also recognised (Hunink and Glasziou, 2001), all *medical* decisions.

## What Is Values-Based Medicine? And Why Do We Need It?

Decisions in medicine are increasingly made against a background of complex and often conflicting values. Again, the most obvious evidence of this is in the growing importance of ethical issues in medicine. But there are many other increasingly “values complex” areas of medicine, such as clinical governance, audit, quality assurance, concerns about cost-effectiveness, and the use of quality-of-life and other similar measures in preventive and public health medicine.

It is against this background of the growing complexity of values in all areas of medicine that the need for “values-based medicine,” as it is increasingly coming to be called, arises. Medicine has of course always been, at least in part, values-based—the Hippocratic “oath,” after all, as the first code of medical ethics, takes us right back to classical Greece. But just as we need evidence-based medicine because of the increasing complexity of the *evidence* underpinning medical decision-making, so, increasingly, do we need values-based medicine because of the increasing complexity of the *values* underpinning medical decision-making.

Thus, we can think of values-based medicine as being to values what evidence-based medicine is to *evidence*. Just as evidence-based medicine offers a process for working more effectively with complex and conflicting evidence in medicine, so values-based medicine offers a process, albeit a different process, for working more effectively with complex and conflicting *values* in medicine.

## What Is Values-Based Practice? And Why Do We Need It?

A wide variety of disciplines are already contributing to values-based medicine. In addition to ethics and law, these include: health economics, decision analysis, the history of medicine, medical humanities, and the social and anthropological sciences.

However, important as the contributions of these disciplines have been, when it comes to clinical decision-making in day-to-day practice, it often seems that we are not being as effective as we should be in joining up values with evidence. This is particularly clear, for example, in feedback from patients and patient groups on clinical guidelines. In this context, ethics has sometimes been seen as being something of a palliative when it comes to applying evidence-based guidelines in practice. Patient autonomy, in particular, is seen as underpinning the patient's right to choose even if what they choose is contrary to the evidence from a medical point of view.

This is where values-based practice has a role to play in strengthening the resources of values-based medicine. Based on work in analytic philosophy (in particular, Fulford, 1989), together with a number of empirical studies (notably, Colombo *et al*., 2003), values-based practice offers a new and primarily skills-based approach to working with complex and conflicting values in medicine. A particular feature of values-based practice is that, as described further below, it is fully complementary to and supports evidence-based approaches. A detailed clinical example, showing the fully complementary way in which evidence-based and values-based approaches work out in practice, is given in Fulford's (2004) account of “the artist who couldn't see colours.” The artist in question, Diane Abbot (not her real name), was a senior academic art historian who had suffered for some years from bipolar disorder. Her story is set out in this chapter in a series of ten episodes that illustrate all ten of the key elements of the process of values-based practice. These include four areas of clinical skills outlined below but also two aspects of service design (that services should be person-centered and multidisciplinary), three key links between evidence and values in clinical decision-making, and a new model of the partnership that is needed between patients and clinicians.

The essence of Diane Abbot's story is the way in which values-based approaches complement evidence-based approaches throughout. Thus, as an academic, Diane Abbot had read up on lithium as a prophylaxis for bipolar disorder and she had also heard good reports of its effects from an academic lawyer and colleague with a problem similar to her own. When she started on lithium she did indeed do very well for several months from a medical point of view: she had few side effects, her lithium levels were well controlled, and the intensity and frequency of her mood swings were greatly reduced. It was something of a surprise, then, to her doctor when she suddenly announced that she had decided to come off lithium. The reason, as she put it, was that she could “no longer see colours.” She did not mean that she had become colour blind! What she meant was that colours had lost their emotional intensity. This was a side effect of lithium that had not been mentioned in the medical literature (though it has subsequently been emphasized in patient narrative, e.g., Jamison, 1994) because the emotional blunting that it caused had not been seen as being important compared with its more obvious medical benefits. But from Diane Abbot's perspective, within her frame of values as an artist, it was absolutely crucial; so she stopped the lithium. Nonetheless, the story had a positive outcome. Through the process of open discussion of values and evidence with her doctor she felt confident enough to allow a particular colleague to take the initiative and to insist on her obtaining short-term treatment with neuroleptics when she appeared to be “going high.” She had not been able to do this previously, but this now formed the basis for effective management.

## The Development Of Values-Based Practice In Mental Health

Values-based practice has been developed mainly in mental health through a number of initiatives, led originally by the Department of Philosophy and more recently by the new Medical School at Warwick University, working with national and international partner organizations representing the three key stakeholder groups of patients and carers, professionals and policy makers.

As described further below, partner organizations in the development of values-based practice have included, in particular, a major mental health NGO in London, The Sainsbury Centre for Mental Health, the UK government's Department of Health, and the World Psychiatric Association. Working together with these and other groups, we have seen significant progress in three key areas of policy, training, and research.

### 1. Policy

A national framework setting out a number of key principles of values-based practice has been developed by the National Institute for Mental Health in England (NIMHE; www.nimhe.org.uk). NIMHE is the section of the UK government's Department of Health that has the responsibility of implementing policy in mental health. As such, the NIMHE Values Framework has subsequently become the basis for a series of specific policy and service development initiatives concerned with improving multidisciplinary and multi-agency teamwork as the basis of more effective patient-centered care (see, e.g., Department of Health, 2004a and 2004b).

### 2. Training

A comprehensive training manual for values-based practice, *Whose Values?* (Woodbridge and Fulford, 2004), developed and piloted with front-line mental health and social care staff, was launched by the Minister of State in the Department of Health, Rosie Winterton, at a conference in London in 2005. The training manual has subsequently been used successfully in a wide variety of clinical contexts and particular sections of it have been further developed in the form of a CD-Rom resource to support the policy initiatives noted above (Woodbridge and Fulford, 2005).

There are four main skills areas of values-based practice each of which draws directly on philosophical sources:

***Awareness:*** The training exercises that help to develop awareness of values, and of the often surprising diversity of values, draw directly on work in the Oxford tradition of linguistic analytic philosophy, including that of R. M. Hare (Hare, 1952, 1963) and also J. L. Austin (Austin, 1956/57). The materials are not presented as philosophy! But it is a mark of the strongly practical relevance of this particular philosophical tradition that it lends itself so readily to the development of skills training exercises for front-line staff.

It is these exercises that form the basis of the training manual, *Whose Values?* (Woodbridge and Fulford, 2004). An example taken from an early section of the manual, and of course normally done as a workshop, runs along the following lines: participants are asked to bring to the workshop a sample of any piece of written material that they use in their day-to-day practice; this might mean, for example, a letter to another doctor, a clinical practice guideline, or a policy statement. In the workshop, participants are asked to go through the text they have brought with them and to highlight values by picking out values-related words. Most participants start by saying that the text is not about values, but what they find by the end of the exercise, and the subsequent discussion, is that many different values are in fact expressed right through the text. This exercise builds on Austin's distinction between reflective definition and the actual *use* of concepts (Austin, 1956/57). When we simply reflect, we are generally unaware of the many values that impinge on our work. They are important but, like the air we breathe, we do not notice them. But when we look carefully at the language we actually use, then we become aware of the range and diversity of values that have been there all the time.

***Reasoning:*** The reasoning skills for values-based practice draw on all the reasoning skills that have been well-developed in philosophical ethics (such as principles reasoning, casuistry, utilitarianism, and deontology) but with a distinctive twist—values-based reasoning is used not to support a particular ethical position but to explore the range of often very different values that may be present in a given situation (Fulford*et al*., 2002).

With principles reasoning, for example, ethical issues around compulsory treatment are normally analyzed by balancing the principle of autonomy (i.e., that patients should be free to choose what happens to them) against that of beneficence (i.e., that doctors should act in the best interests of their patients). This can be very helpful in resolving ethical dilemmas. But principles reasoning, as fully set out by Beauchamp and Childress (1989/1994), includes two further important principles—of justice (equal treatment) and non-maleficence (avoiding harm)—and these can help to reveal hidden aspects of a case. Thus, in the case of compulsory treatment, values relating to equality of treatment, although important, are often neglected: issues of resources (or the lack of them) are often extremely important here although rarely noted, particularly by lawyers! Again, the principle of non-maleficence often exposes hidden but key issues about risks of harm to the patient or others that may not have been thought about. Thus, the four principles, taken together, are like four key dimensions along which the values-aspects of a case can be comprehensively explored.

***Knowledge:*** One of the links between values-based and evidence-based approaches is that values-based practice is, as far as possible, evidence-based! Many people think that values can just be taken for granted. But it is recognized in values-based practice that it is just as important to “listen to the evidence,” rather than rely on intuition alone, when finding out about values as it is in the more traditional areas of evidence-based practice such as the effectiveness of different treatment options.

There are many ways of getting information regarding values. These include, for example, personal narratives of patients and family carers and a number of powerful philosophical methods—phenomenology (Stanghellini, 2004), hermeneutics (Widdershoven and Widdershoven-Heerding, 2003), and discursive philosophy (Sabat, 2001)—and directly empirical methods (Colombo *et al*., 2003).

These different ways of getting knowledge about values are of course complementary. Thus, among the examples noted above, phenomenological and hermeneutic methods both provide powerful skills-based methods for exploring unfamiliar areas of people's experience and behaviour, where these are the basis of psychopathology. Phenomenological methods, building on the work of Karl Jaspers (1913), are of course the basis of much of modern descriptive psychopathology. Other approaches that are proving fruitful include Merleau-Ponty's phenomenology and new understanding of the way time is structured for people with dyslexia (Philpott, 1998); and the use of Sartre's detailed phenomenology of the body to characterize the different forms of body dysmorphophobia (Morris, 2003). Similarly, hermeneutics is now providing a powerful tool for exploring the experience of people with Alzheimer's disease (Widdershoven and Widdershoven-Heerding, 2003); and it is in this area too, and again in complementary ways, that the theory and skills of discursive psychology (that explores how meaning is created through discourse) are also proving extremely powerful (Sabat, 2001).

***Communication skills:*** Communication skills are central to effective decision-making in values-based practice and, in particular, for bringing evidence and values together in individual cases (Hope *et al*., 1996). This is an area where methods and resources from management theory may be particularly helpful, both for exploring differences of values and for resolving conflicts between values (Fulford and Benington, 2004).

In addition to general communication skills, there are particular skills needed to support both person-centered care and multidisciplinary teamwork. The empirical study by Colombo *et al.*, (2003), noted above, illustrates the importance of this. In essence, the study employed a combination of analytic philosophical and empirical social science methods (Fulford and Colombo, 2004) to show that, although different members of multidisciplinary teams (psychiatrists, social workers, nurses, etc.) thought that they all had the same shared values, in practice they were often being driven by very different values: for example, doctors were more concerned about medication and social workers were more concerned about risk. So long as they were unaware of them, these differences of values were a source of failures of communication and of (unaccountable, at the time) difficulties in teamwork and shared decision-making. Once team members were aware of these differences, by contrast, they became the basis for a person-centered approach to each patient or client, in which different aspects of their individual situation could be balanced appropriately. So the hidden values, once made explicit, were changed from being a problem to an asset!

A recent and particularly exciting training development in the UK is the production of targeted values-based materials to support implementation of a new Mental Health Act. The Act covers the use of compulsory treatment in psychiatry. This is a particularly key area for values-based practice because compulsion by its very nature involves a direct conflict of values between the patient concerned and everyone else.

This work, which is being led by Malcolm King, one of the trainers who contributed to the development of the original training manual, *Whose Values?* (Woodbridge and Fulford, 2004), uses the “guiding principles” behind the Act to support how the Act is applied in practice. The basic approach is that the law is the starting point — it is the law that tells us *what* to do. There is then a “code of practice” that tells us *how to apply the law in general*. This code of practice is really a guide for clinicians and other non-lawyers. But it is the guiding principles that, literally, guide us in applying the law and the code of practice *in individual situations*. This is because they represent a set of the key values (such as respect, good communication, assessment of risk, etc.) that need to be balanced when coming to particular decisions about compulsion in individual situations.

***Research:*** The research frontier for values-based practice is diagnosis. In medicine, we tend to think that diagnosis is an area of decision-making that is “purely” scientific and hence value-free. However, when it comes to applying a number of key diagnostic criteria in psychiatry, it is clear that diagnostic assessment depends not only on the facts (the evidence we are presented with) but also on a number of value judgments that we make based on those facts. This is clear, for example, in the case of the criteria of clinical significance in the American Psychiatric Association's Diagnostic and Statistical Manual (DSM): the judgments of dysfunction on which these criteria depend are essentially value judgments—for example, it is a matter of individual and cultural values whether working very long hours at the expense of your family life is a “good” thing; similarly, someone who leaves his home and work and goes off to meditate in the desert maybe thought to be functioning well in a society that values religious retreat, but to be functioning badly in a society that is more work-oriented. So values are important here, at the very heart of the DSM, but they are also present throughout the DSM and, indeed, other major psychiatric classifications (Sadler, 2005).

NIMHE, working in partnership with a number of international organizations, including both the World Psychiatric Association and the Mental Health and Substance Abuse Section of the WHO, has supported a series of international research seminars that have brought together clinicians, patients, and policy makers to explore the role of values in psychiatric assessment and diagnosis. These seminars have culminated in a national consultation by the Department of Health, the aim of which is to develop a model of assessment that brings together the different approaches of different professional groups within the multidisciplinary team in a shared process with the individual service user concerned (see consultation document at http://www.dh.gov.uk/en/Consultations/Liveconsultations/DH_080913).

The skills-based approach of values-based practice has thus proved highly successful in mental health and social care in the UK through these and other policy, training, and research initiatives.

There have also been important developments internationally. The World Psychiatric Association, through its Institutional Program on Psychiatry for the Person (IPPP) (Mezzich and Salloum, 2007), has been particularly active in this area with a number of key initiatives, including important innovations in person-centered diagnosis (Mezzich, 2007). There have also been significant developments in a number of Continental European countries and in South Africa (Van Staden and Fulford, 2007). The person-centered approach and more traditional objective diagnostic criteria, it is important to add, are fully complementary. The objective criteria provide the basis for general categorizations related to evidence of effectiveness of treatment, epidemiological data, and so forth. But these general categories need to be related to individuals and the particular circumstances in which they find themselves, and it is here that a more person-centered approach becomes important. This can be thought of in terms of the traditional distinction between diagnosis and “formulation” around the individual case.

## Conclusions—Psychiatry First!

As noted at the start of this editorial, values-based practice, although a *skills*-based approach to working with complex and conflicting values, is at the practical cutting edge of a new interdisciplinary field involving philosophy and psychiatry.

To many, it came as a considerable surprise that the new field of philosophy of psychiatry should have emerged when it did, in the 1990s, heralded as this decade had been as the “decade of the brain” (Fulford *et al*., 2003). There has been important work in the philosophy of psychiatry at earlier periods, of course, not least in the foundational work of Karl Jaspers (Jaspers, 1913). But the widespread expectation had been that with the development of the new neurosciences, the need for philosophy in psychiatry would diminish rather than increase (Fulford *et al*., 2006). As Singh (2007) has put it “While the experimental breakthroughs, both in etiology and therapeutics, will come mainly from biology, the insights and leads can hopefully come from many other fields, especially the psychosocial and philosophical. It is in some such synergy that these two supposedly antagonistic branches must engage themselves, to complement and nurture rather than confront and dismember.”

So, nothing could be further from the truth than the idea that philosophy and neuroscience run counter to each other! Indeed, as the American neuroscientist and psychiatrist Nancy Andreasen (Andreasen, 2001) has argued, developments in the neurosciences, far from reducing the need for rigorous philosophical work in psychiatry, actually drive many of the deepest problems of traditional philosophy to the very top of our agenda—such problems include the nature of personal identity, the problem of free will, and the problem of the relationship between mind and brain itself.

When it comes to values, in particular, there are many reasons why work on the philosophy of values, and its practical counterpart in values-based practice, should have been pushed to the top of our agenda. Some of these reasons are to do with changes in society as a whole—the increasingly multicultural nature of society, for example, and the ever more diverse cultural and social values this brings with it. Other more directly medical reasons have to do with changes in professional practice—the extension of multidisciplinary team working, for example, with different clinical disciplines bringing, often, very different sets of professional values to the clinical encounter.

The most important reason, however, for the increasing importance of values in medicine has to do with the emergence of a model of patient-centered practice in which the values of individual patients are central to evidence-based clinical decision-making. Sackett *et al*., from whose book on evidence-based practice I quoted at the start of this editorial, underline the importance of this, writing that it is only” (w)hen these *three* elements (best research evidence, clinical expertise, and patient values) are integrated, (that) clinicians and patients form a diagnostic and therapeutic alliance which optimizes clinical outcomes and quality of life (Sackett *et al*., 2000, page 1, emphasis and parentheses added).

Psychiatry, therefore, in being first in the field with policy, training, and research developments in values-based practice as an essential partner to evidence-based practice, is leading the way towards a medicine for the 21st century that is both firmly science-based and also genuinely patient-centered.[33]

### Take Home Message

This editorial has illustrated some of the ways in which work in the new interdisciplinary field of philosophy of psychiatry—particularly as developed through the skills-based approach to working with complex and conflicting values called values-based practice—is complementary to, and supports developments in, evidence-based practice in providing clinical care in psychiatry that is equally science-based and person-centered.

## About the Author

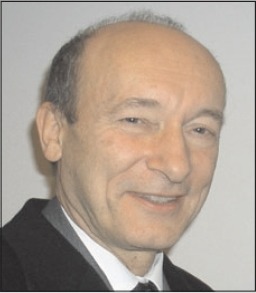



Prof K.W.M. Fulford, Dphil, FRCP, FRCPsych is Professor of Philosophy and Mental Health, University of Warwick; Member of the Philosophy Faculty, Honorary Consultant Psychiatrist, and Fellow of St Cross College, University of Oxford; Co-Director, Institute for Philosophy, Diversity and Mental Health, Centre for Ethnicity and Health, University of Central Lancashire; Editor, Philosophy, Psychiatry, & Psychology; Special Advisor for Values-Based Practice, Department of Health, London; and is on the Honorary International Editorial Advisory Board of the Mens Sana Monographs.
